# Expression of mutant mRNA and protein in pancreatic cells derived from MODY3- iPS cells

**DOI:** 10.1371/journal.pone.0217110

**Published:** 2019-05-30

**Authors:** Shigeharu G. Yabe, Junko Nishida, Satsuki Fukuda, Fujie Takeda, Kiyoko Nasiro, Kazuki Yasuda, Naoko Iwasaki, Hitoshi Okochi

**Affiliations:** 1 Department of Regenerative Medicine, Research Institute, National Center for Global Health and Medicine, Tokyo, Japan; 2 Department of Metabolic Disorders, Diabetes Research Center, National Center for Global Health and Medicine, Tokyo, Japan; 3 Institute of Geriatrics, Diabetes Center, Institute of Medical Genetics, Tokyo Women’s Medical University, Tokyo, Japan; Osaka Shiritsu Daigaku, JAPAN

## Abstract

Maturity-onset diabetes of the young (MODY) is a heterozygous monogenic diabetes; more than 14 disease genes have been identified. However, the pathogenesis of MODY is not fully understood because the patients’ pancreatic beta cells are inaccessible. To elucidate the pathology of MODY, we established MODY3 patient-derived iPS (MODY3-iPS) cells using non-integrating Sendai virus (SeV) vector and examined the mutant mRNA and protein of HNF1A (Hepatocyte Nuclear factor 1A) after pancreatic lineage differentiation. Our patient had a cytosine insertion in the HNF1A gene (P291fsinsC) causing frameshift and making a premature termination codon (PTC). We confirmed these MODY3-iPS cells possessed the characteristics of pluripotent stem cells. After we differentiated them into pancreatic beta cells, transcripts of HNF1A gene were cloned and sequenced. We found that P291fsinsC mutant transcripts were much less frequent than wild ones, but they increased after adding cycloheximide (CHX) to the medium. These results suggested that mutant mRNA was destroyed by nonsense-mediated mRNA decay (NMD). Moreover, we were not able to detect any band of mutant proteins in pancreatic lineage cells which were differentiated from MODY3-iPSCs by western blot (WB) analysis. A scarcity of the truncated form of mutant protein may indicate that MODY3 might be caused by a haplo-insufficiency effect rather than a dominant negative manner.

## Introduction

Maturity-onset diabetes of the young (MODY) is an autosomal dominant form of monogenic diabetes that stems from one or more mutations in a single gene. To date, 14 disease genes for MODY are identified; most of them are transcription factors MODY1 (*HNF4A*), MODY3 (*HNF1A*), MODY4 (*PDX1*), MODY5 (*HNF1B*), MODY6 (*NEUROD1*/*BETA2*), MODY7 (*KLF11*), MODY8 (*CEL*), MODY9 (*PAX4*), MODY11 (*BLK*), and MODY14 (APPL1). Disease genes also encode enzyme MODY2 (*GCK*), hormone MODY10 (*INS*) and channel protein MODY12 (*ABCC8*), MODY13 (*KCNJ11*) [1, 2]. Although MODY is considered to be a genetic disease, it is noteworthy that diabetic phenotype appears usually in teens or older, excluding MODY2 which is caused by mutations in glucokinase gene that codes glycolytic enzyme. The pathogenesis of each MODY is characterized by dysfunction of the pancreatic beta cells; however, precise mechanisms are not fully understood. Until recently, it has been very difficult to elucidate the molecular mechanisms underlying MODY because pancreatic beta cells from the patients were not available for experiments due to ethical issues.

Successful generation of induced pluripotent stem cells (iPSCs) from somatic cells drastically improves the situation for investigating pathological mechanisms of genetic diseases [3, 4]. For example, primary human neuronal cells from Parkinson’s disease patients have not been available for research; however, this new methodology made it possible to analyze the pathogenesis of Parkinson’s disease by differentiating patient-derived iPSCs into neurons [5, 6]. Such disease specific iPSCs enable us to reproduce a disease model system in vitro. Today, the number of reports of generating disease specific iPSCs is increasing [7–9], and establishment of MODY patient derived-iPSCs and differentiation to the pancreatic lineage have been reported (MODY-iPSCs) [10–14]. These MODY-iPSCs were generated mainly from Caucasian patients using retro virus, Cre-excisable polycistronic lentivirus, or non-integrating Sendai virus. We previously reported establishment of MODY5-iPSCs from a Japanese patient induced by non-integrating Sendai Viruses. Because the capacity for insulin secretion is larger in Caucasians than in Asians [15], the genetic background should be considered when investigating the pathophysiology of diabetes. Therefore, establishment of MODY-iPSCs from various populations is vital for analyzing the pathogenesis of MODY.

Although 14 MODY disease genes have been reported, each of them has many different mutation sites [16–18]. For example, among MODY3 patients, more than 200 mutation sites have been found in the HNF1a gene [17]. Several papers suggest that different mutations lead to different types of the mutant protein activity, including loss-of-function or dominant-negative activity [19–22]. In this study, we chose a MODY patient with P291fsinsC mutation because MODY3 is the most common type among MODY and P291fsinsC is the most common mutation pattern of MODY3 [18, 23–26]. Moreover, P291fsinsC mutation in the HNF1A gene had a cytosine insertion in the fourth exon causing a frameshift and creating a premature termination codon (PTC) before a normal stop codon. PTCs are usually caused by frameshift or nonsense mutations [27, 28] and theoretically, PTC-bearing mRNA is supposed to make a C-terminal truncated protein. In case of MODY3, there is controversy regarding the roles of mutant proteins. Artificially synthesized mutant proteins were reported to act in a dominant negative manner by in vitro experiments [19, 21]. Another group also reported that mutant MODY3 mRNA having PTC was degraded by nonsense-mediated mRNA decay (NMD) and suggested the haplo-insufficiency effect [29]. They proved NMD by ectopic transcripts made from patient-derived lymphoblastic cells transformed by Epstein-Bar virus. Therefore, it is important to examine whether or not mutant protein has dominant-negative activity in the patient pancreatic beta cells. In this research, we established MODY3-iPSCs from Japanese patients using non-integrating Sendai virus vector and examined their mutant mRNA and protein of HNF1A after pancreatic lineage differentiation.

## Methods

### Generation of MODY3-iPSCs

Generation of MODY-iPS was based on our previously reported protocol [30]. Skin fibroblasts from a MODY3 (P291fsinsC) patient were obtained by 5 mm punch biopsy at Tokyo Women’s Medical University after written informed consent. Aliquots of 10^6^ cells of MODY3-patient skin fibroblasts were transduced with *hSOX2*, *hOCT3/4*, *hKLF*, and *hC-MYC* using SeV vectors (MBL, Nagano, Japan) overnight. These cells were washed and cultured in DMEM supplemented 10% fetal bovine serum (FBS) for 6 days. Then SeV-infected fibroblasts were seeded on mitomycin (MMC; Wako, Osaka, Japan) treated mouse embryonic fibroblast (MEF) feeder cells. The next day, the medium was replaced by hiPS medium (DMEM/F’12 supplemented with 20% Knockout serum replacement (KSR; GIBCO BRL, Palo Alto, CA, USA), 2 mM L-glutamine (Wako), 0.5x penicillin/ streptomycin (Wako), 1x non-essential amino acids (Wako), 55 μM 2-mercaptoethanol (Gibco) and 7.5 ng/ml FGF2 (Peprotech, Rocky Hill, NJ, USA)). Three to four weeks later, primary hiPS colonies appeared. We transferred each colony onto MMC treated SNL feeder cells (ECACC, Salisbury, UK). These hiPS colonies were maintained on MMC treated SNL feeder cells and passaged using CTK solution at 1:5–1:8 once a week as described previously [31]. Control -iPSCs had previously been established from healthy donor fibroblasts [30]. These experiments were carried out with the approval of ethical committees in Tokyo Women’s Medical University and in National Center for Global Health and Medicine.

### Differentiation protocol

For sequencing analysis, differentiation from hiPS cells into pancreatic beta cells was based on the Maehr’s method [32] with minor modifications [30] using adherent culture.

For protein analysis, we differentiated pancreatic beta cells using our suspension culture methods [33]. Dissociated MODY3-iPSCs were seeded into ultra-low attachment 6-well plates at a density of 10^6^ cells/ml in 4ml Essential 8 medium (Gibco) including 10 μM Y-27632 (Cayman Chemical, Ann Arbor, MI, USA) on orbital rotators set at 90 rpm. After overnight culture, the medium was exchanged. The next day, Essential 8 was replaced with hiPS medium for 1 day; then DE induction was initiated. At stage 1 (DE: definitive endoderm), spheroids were cultured for 4 days in RPMI 1640 (Wako) supplemented with 0.25% bovine serum albumin (BSA; Sigma, USA), 0.4x penicillin and streptomycin (PS; Wako), 1 mM sodium pyruvate (Wako), 1x NEAA, 80 ng/mL recombinant human activin A (Peprotech) and 55 μM 2-ME. Fifty ng/ml FGF2, 20 ng/ml recombinant bone morphogenetic protein 4 (BMP4; Peprotech) and 3 μM CHIR99021 (Biovision, Milpitas, CA, USA) were added for the first 2 days, and 0.5% KSR was added on Day 4.

At stage 2 (PGT: primitive gut tube), spheroids were cultured for 3 days in RPMI 1640 supplemented with 0.25% BSA, 1 mM sodium pyruvate, 1x NEAA, 0.4x PS, and 50 ng/ml recombinant human FGF7 (Peprotech), 1% B27 supplement (GIBCO) and 1:333 insulin, transferrin, selenium, ethanolamine solution (ITS-X; Gibco). The medium was changed on the third day.

At stage 3 (PFG: posterior fore gut), spheroids were cultured in DMEM (8mM glucose) supplemented with 0.15% BSA, 0.4x PS, 1x NEAA, 50 ng/ml FGF7, 1% B27 supplement, 1:333 ITS-X, 0.5 μM EC23 (Santa Cruz Biotechnology), 0.2 μM LDN 193189 (Cayman Chemical), 0.3 μM indolactam V (ILV; Cayman Chemical), and 0.25 μM SANT1 (Cayman Chemical) for 4 days. The medium was changed every 2 days during stage 3.

At stage 4 (PP: pancreatic progenitor), spheroids were cultured in DMEM (8 mM glucose) supplemented with 0.15% BSA, 0.4x PS, 1x NEAA, 50 ng/ml recombinant human FGF10 (Peprotech), 1% B27 supplement, 1:333 ITS-X, 0.04 μM EC23, 0.2 μM LDN 193189, 0.3 μM ILV, and 0.25 μM SANT1, 10 μM Alk5 inhibitor II (RepSox; Biovision) and 5 μM ZnSO_4_ (Sigma) for 3 days. The medium was changed on the third day.

At stage 5 (EP: endocrine progenitor), spheroids were cultured in DMEM (20 mM glucose) supplemented with 0.15% BSA, 0.4x PS, 1x NEAA, 20 ng/ml recombinant human epidermal growth factor (EGF; Peprotech), 1% B27 supplement, 1:333 ITS-X, 0.02 μM EC23, 0.2 μM LDN 193189, 0.25 μM SANT1, 10 μM Rep Sox, 5 μM ZnSO_4_, 50 ng/ml exendin-4 (Abcam), 10 μg/ml heparin (Sigma), 10 μM Y27632, 0.5 μM DBZ (Cayman Chemical) and 5 mM Nicotinamide (Sigma) for 7 days; the medium was changed every 2 days during stage 5.

At stage 6 (BETA: beta cell stage), spheroids were cultured in DMEM (20 mM glucose) supplemented with 0.15% BSA, 0.4x PS, 1x NEAA, 1% B27 supplement, 1:333 ITS-X, 10 μM Rep Sox, 5 μM ZnSO_4_, 50 ng/ml exendin-4, 1 μM R428 (Cayman chemical), 10 μg/ml heparin, 5 mM Nicotinamide, 10 ng/ml BMP4, 50 ng/ml recombinant human hepatocyte growth factor (HGF; Peprotech), 50 ng/ml insulin-like growth factor 1 (IGF-1; Peprotech) and 5 μM forskolin (Wako) for 10 days. The medium was changed every 2 days during stage 6.

### CHX treatment

MODY3-iPSCs were treated with 0.25 mM cycloheximide (CHX) for 18hr at the beta stage.

### Alkaline phosphatase staining and immunocytochemistry

Alkaline phosphatase staining was performed using the Leukocyte Alkaline Phosphatase kit (MUTO PURE CHEMICALS, Tokyo, Japan). Immunocytochemistry of pluripotency markers was performed as described previously [30]. MODY3-iPSCs were fixed with 4% paraformaldehyde at RT for 20 min. For the detection of SeV, these cells were fixed using ice cold methanol for 10 min. Fixed cells were rinsed with phosphate buffered saline (PBS), permeated with PBS containing 0.3% Triton X-100 for 15min, and blocked with PBS containing 0.1% tween20 and 3% bovine serum albumin (BSA) for 1hr. Then the cells were incubated overnight at 4°C with the following primary antibodies: mouse anti-Oct3/4, 1:100 (611203, BD, Mountain View, CA); rabbit anti-Nanog, 1:100 (RCAB0003P, Reprocell, Kanagawa, Japan); mouse anti-SSEA4, 1:50 (90231, Millipore, Temecula, CA); moues anti-Tra-1-60,1:50 (90232, Millipore); mouse anit-Tra-1-81, 1:50 (90233, Millipore); rabbit anti-SeV, 1:500 (PD029, MBL). After incubation, cells were washed with PBS containing 0.1% tween20 and incubated for 2hr at RT with Alexa Fluor 594-conjugated donkey anti-mouse IgG (A-21203, Invitrogen, CA, USA) or Alexa Fluor 594-conjugated donkey anti-rabbit IgG secondary antibodies (A-21207, Invitrogen). Immunocytochemistry of beta cells marker was carried out as described previously [28]. Cultured spheroids were observed every day under an IX71 microscope (Olympus, Tokyo, Japan). Spheroids were collected during and after the differentiation process, embedded in Optimal Cutting Temperature (OCT) compound (Sakura Fintek Japan), and stored at -80°C. Then 6-μm cryosections were cut and immunostained using the following antibodies: goat anti-PDX1/IPF-1, 1:00 (AF2419, R&D), mouse anti-NKX6.1, 1:00 (F55A12-s, DSHB, University of Iowa), rat anti-C-peptide, 1:200 (GN-ID4-s, DSHB), rabbit anti-HNF1A, 1:1000 (89670, CST). The following secondary antibodies were used respectively: Alexa Fluor 488-conjugated donkey anti-goat IgG (A-11055; Invitrogen), Alexa Fluor 488-conjugated donkey anti-mouse IgG (A-21202; Invitrogen), Alexa 594-conjugated goat anti-rat IgG (A-11007; Invitrogen) and Alexa Fluor 488-conjugated goat anti-rabbit IgG (A27034; Invitrogen). Slides were counterstained with 4′,6′-diamidino-2-phenylindole (DAPI; Invitrogen) prior to mounting with Fluoromount (Diagnostic Biosystems).

### Teratoma assay

Animal studies were conducted according to protocols approved by the Animal Care and Use Committee in the National Center for Global Health and Medicine. Eight-week old NOD-SCID mice (JAPAN Clea) were used for experiments. Undifferentiated MODY3-iPS cells (one x 10^6^ cells) were grafted into a testis (under the capsule) of SCID mice anesthetized with Sevoflurane. After 10–12 weeks, teratomas were extirpated and processed according to standard protocol for paraffin embedding and hematoxylin and eosin staining.

### Karyotype analysis

Three different colonies of MODY3-iPSCs were picked up, and, over the 10th passage, each cell line containing 1 x 10^6^ cells was sent to MBL Research Laboratories, Japan, where chromosomal G-band analysis was performed.

### Cloning and sequencing

Total RNA was extracted and purified from pancreatic beta cells differentiated-MODY3-iPSCs with Isogen (Wako, Shiga, Japan). cDNA was synthesized with random nonamer and oligods (dT18) using Prime Script II reverse transcriptase (Takara Bio, Osaka, Japan). RT-PCR was performed with GoTaq DNA polymerase (Promega, Madison, WI, USA). Amplification of *HNF1A* cDNA was carried out by RT-PCR with the following primers: *HNF1A*, a forward primer (5’-GCTAGTGGAGGAGTGCAATAGG-3’) and a reverse primer (5’-CTTGGCTTCTGTACTCAGCAGG-3’). Amplification of *HNF1A* genomic fragment was conducted by genomic PCR with following primers: HNF1A, a forward primer (5’-GTGGAGGAGTGCAATAGGTACAAC-3’) and a reverse primer (5’-CCACATACCACTTACCGTGGAC-3’). These amplicons were purified with Wizard SV Gel and PCR Clean-UP System (Promega) and direct sequences were performed at MACROGEN, Japan. To detect transgenes by SeV genome RT-PCR was performed using following primers: SeV genome, a forward primer (5’-GGATCACTAGGTGATATCGAGC-3’) and a reverse primer (5’-ACCAGACAAGAGTTTAAGAGATATGTATC-3’), OCT3/4, a forward primer (5’-CCCGAAAGAGAAAGCGAACCAG-3’) and a reverse primer (5’-AATGTATCGAAGGTGCTCAA-3’), SOX2, a forward primer (5’-ACAAGAGAAAAAACATGTATGG-3’) and a reverse primer (5’-ATGCGCTGGTTCACGCCCGCGCCCAGG -3’), KLF4, a forward primer (5’-ACAAGAGAAAAAACATGTATGG-3’) and a reverse primer (5’-CGCGCTGGCAGGGCCGCTGCTCGAC-3’), c-MYC, a forward primer (5’-TAACTGACTAGCAGGCTTGTCG-3’) and a reverse primer (5’-TCCACATACAGTCCTGGATGATGATG-3’).

### Vector construction

Wild and mutant constructs of *HNF1A* (NM_001306179) and *HNF1B* (NM_000458) were artificially synthesized by GenScript (Piscataway, USA Piscataway, NJ 08854 USA).

### Quantitative RT-PCR

Total RNA was isolated and purified from pancreatic beta cells differentiated-MODY3-iPSCs using Isogen (Wako). The cDNA synthesis was performed with PrimeScript II reverse transcriptase using random nonamers and oligos (dT18). Quantitative RT-PCR reactions were conducted on CFX96 Touch Deep Well (Bio-Rad, Hercules, CA, USA) using GoTaq qPCR master mix (Promega). Relative quantification was performed against a standard curve, and the expression levels of target genes were normalized against that of the housekeeping gene, ornithine decarboxylase antizyme (OAZ1). Primers sequence; HNF1A, a forward primer (5’-TCC CTT AGT GAC AGT GTC TAC ACC-3’) and a reverse primer (5’-AGA CCA GCT TGG CTT CTG TAC TC-3’). OAZ1, a forward primer (5’-GTC AGA GGG ATC ACA ATC TTT CAG-3’) and a reverse primer (5’-GTC TTG TCG TTG GAC GTT AGT TC-3’).

### Western blotting

Differentiated cells were collected in RIPA Buffer (Wako) with protease inhibitors (Roche, Basel, Switzerland), homogenized, and centrifuged. Concentration of whole cell lysate protein was determined by a BCA protein assay (TakaRa). Proteins were boiled in sample buffer (BIO-RAD) and loaded onto Mini-PROTEAN TGX Gels (BIO-RAD) separated by SDS-PAGE. Protein bands were transferred to Trans-Blot Turbo membranes (BIO-RAD), which were then immersed in TBST containing the primary antibody. Then the membranes were treated with Blocking One (Nacalai Tesque, Kyoto, Japan). After washing, the membranes were next immersed in the secondary antibody and reacted with Super Signal West Femto Maximum Sensitivity Substrate (Thermo Fisher Scientific, Waltham, MA). The bands were detected on LAS 4000 mini (Fuji Film, Tokyo, Japan). Western blotting was carried out with anti-HNF1A, 1:1000 (89670, CST), anti-HNF1β, 1:1000 (12533-1-AP, Proteintech) and anti-GAPDH,1:5000 (MA5-15738, Thermo Fisher Scientific). Horseradish peroxidase–conjugated second antibody (1:2000) was purchased from Santa Cruz Biotechnology (sc-2004, Santa Cruz, CA).

## Results

### Establishment of MODY3-iPSCs

**Fig 1:**
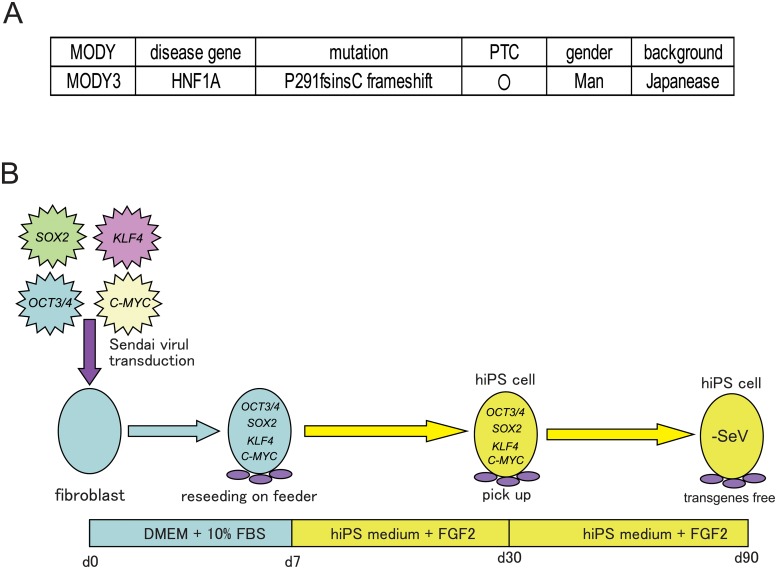
Summary of Japanese patients with MODY3 (P291fsinsC) and the methods of establishment of MODY3-iPS cells. (A) Information about the Japanese MODY3 (P291fsinsC) patient. (B) Time schedule of iPS cell generation using SeV.

**Fig 2:**
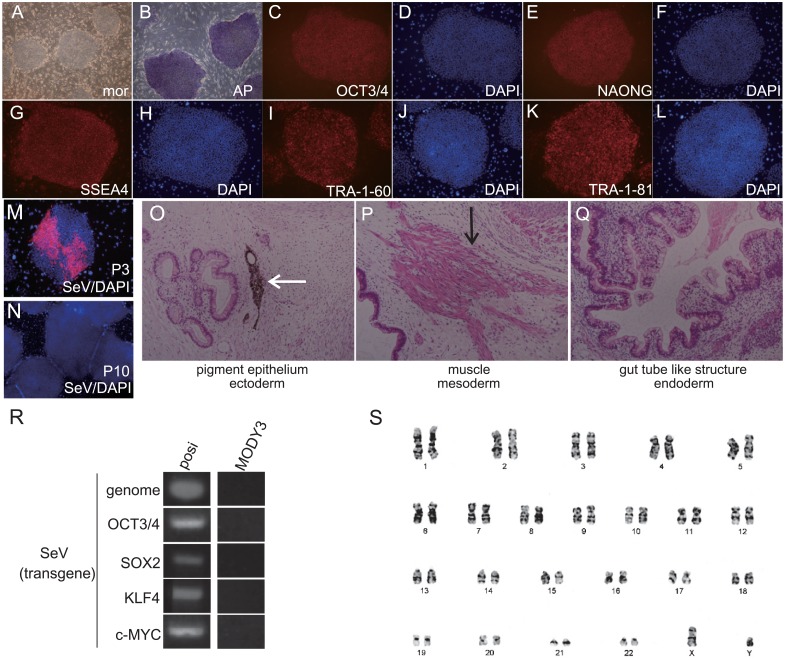
Characterization of pluripotency of MODY3-iPSCs. (A) Representative phase-contrast image of MODY3-iPSCs colonies. mor: morphology of iPSCs. (B) Positive image of alkaline phosphatase (AP) staining. (C-L) Immunocytochemistry for undifferentiated pluripotency markers. (C):OCT3/4, (E):NANOG, (G):SSEA4, (I): TRA-1-60, (K):TRA-1-81, (D,F,H,J,L):DAPI, (M, N) Immunocytochemistry for SeV. (M):passage 3, (N):passage 10 (O-Q): teratoma derived from MODY3-iPS cells (O):ectoderm; pigment epithelium, (P):mesoderm; muscle cells, (Q):endoderm; gut-tube-like structure. White arrow indicates pigment epithelium. Black arrow indicates muscle cells. (R) RT-PCR analysis of transgenes by SeV vector. Posi: positive control (SeV infected-fibroblast cells), MODY3: MODY3-iPSCs. (S):karyotype analysis by G-band method.

Genetic profiles of the MODY3 patient are shown in Fig 1A. To establish transgene free MODY3-iPSCs, patient-derived skin fibroblasts were transduced with *SOX2*, *OCT3/4*, *KLF4* and *C-MYC* using non-integrating Sendai virus. Four weeks later, MODY3-iPS colonies emerged (Fig 1B) and we picked up several colonies and passaged them. Although the SeV genome was present in the cytoplasm of MODY3-iPS colonies at passage 3, no more SeV genome was detected at passage 10 by immunocytochemistry and RT-PCR (Fig 2M, 2N and 2R), indicating that these MODY3-iPSCs were transgene–free. Next, MODY3-iPSCs were characterized by immunocytochemistry and alkaline phosphatase staining. MODY3-iPSCs expressed the undifferentiated pluripotency markers OCT3/4, NANOG, SSEA4, TRA-1-60, and TRA-1-81 and had alkaline phosphatase activity (Fig 2B–2L). To assess the ability to form teratomas, three different cell lines from MODY3-iPSCs were injected into the testes of SCID mice. Large tumors developed three months later. Histological analysis revealed that all the tumors contained various tissues derived from all three germ layers; ectoderm, mesoderm and endoderm (Fig 2M–2O). We confirmed that the karyotype of MODY3-iPSCs was normal (Fig 2S).

### Transcripts with PTC are destroyed in differentiated MODY3-iPSCs

**Fig 3:**
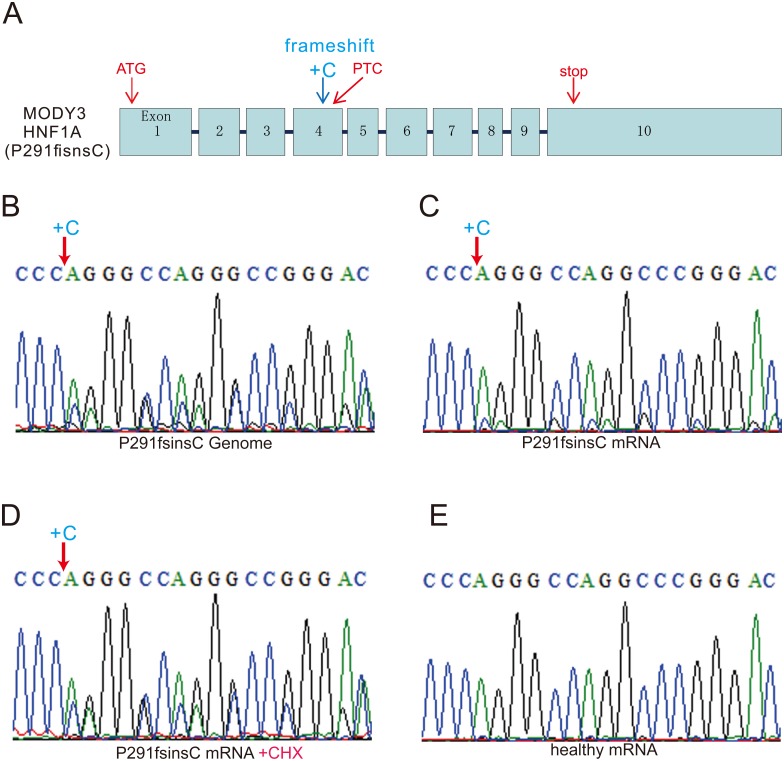
Sequences of wild and P291fsisnC mutant mRNA. (A) Genomic structure of *HNF1A* in the MODY3 patient. The original start and stop codons exist in exon1 and 10, respectively. In the P291fsincC mutation, a frameshift due to the insertion of cytosine brought a new PTC within s exon 4. (B-E) Sequence data for the *HNF1A* gene and transcripts of MODY3-iPSCs and healthy control. (B):Genomic sequence data for the MODY3-iPSCs near the mutation site. (C):Sequence data cloned from transcripts derived from MODY3-iPSCs. (D):Sequence data cloned from transcript derived from cycloheximide (CHX)-treated MODY3-iPSCs. (E) Sequence data cloned from a transcript derived from healthy cells.

In our MODY3 case, a P291fsinsC frameshift mutation of the *HNF1A* gene was the same as reported previously [26]. To detect the mutant mRNA in MODY3-iPS derived cells, we differentiated MODY3-iPSCs into pancreatic lineage cells, cloned mutant mRNA by RT-PCR and then sequenced them. Because the P291fsinsC mutation causes a frameshift by the insertion of cytosine, the genomic sequence data should have both the clear-cut signal of the wild type and a one-base-shifted same pattern signal from the mutation site to the 3’ end. We confirmed this, as shown in Fig 3B. In contrast to the genomic sequence, the strong wild type signal and the faint one-base-shifted mutant mRNA signals were detected in pancreatic lineage cells from MODY3-iPSCs (Fig 3C). These data indicate that the P291fsinsC mutant mRNA was destroyed in differentiated MODY3-iPSCs. We confirmed that the sequence of *HNF1A* mRNA derived from healthy-iPSCs had only clear wild type transcript signals (Fig 3E).

### Restoration of mRNAs by inhibiting NMD with cycloheximide treatment

Degradation of transcripts with PTC in vivo by a NMD pathway known as RNA surveillance system has been suggested. Cycloheximide (CHX) is regarded as an inhibitor of NMD [34]. When Ullrich disease fibroblasts were treated with CHX, they restored collagen VI a2 mutant transcripts containing PTC [35]. We thought that the low amounts of MODY3 mutant mRNAs might be caused by NMD. To confirm this, differentiated MODY3-iPSCs were treated with CHX to inhibit NMD for 18hr before collection of the cells. CHX treatment clearly enhanced the sequence signal of P291fsinsC mutant mRNAs compared with non-treated samples (Fig 3D). We then tried to quantify the expression level of mutant RNAs before and after CHX treatment. Although we designed several taq-man probes against P291fsinsC, this attempt was not successful, because none of the tested taq-man probes could distinguish between wild and mutant mRNAs.

### MODY3-iPSCs were differentiated to pancreatic beta cells using suspension culture

**Fig 4:**
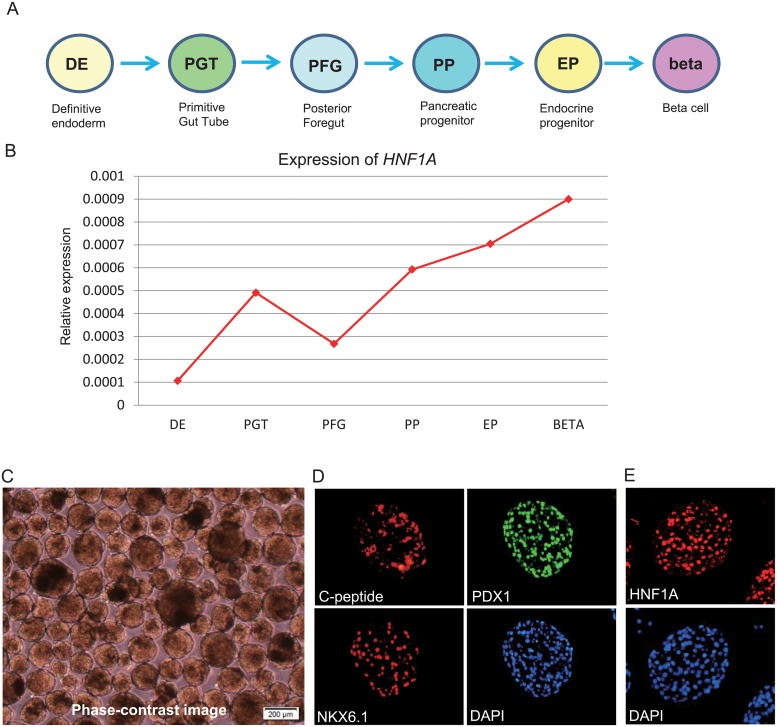
Differentiation of MODY3-iPSCs into pancreatic beta cells. (A) scheme of 6 step differentiation protocol (B), expression pattern of *HNF1A* mRNA during differentiation process, (C) Phase-contrast image of spheroids of differentiated MODY3-iPS-beta cells, (D, E) Immunocytochemistry of differentiated MODY3-iPS-beta cells for pancreatic beta cell markers (D) C-peptide, PDX1, NKX6.1 and DAPI staining, (E) HNF1A and DAPI staining.

As P291fsinsC mutant mRNA was destroyed by NMD, we further examined the mutant gene expression at the protein level. We first tried to check the HNF1A protein by western blot using a previously reported adherent culture protocol [30, 31], but could not detect it. One possible reason is that differentiation efficiency was not enough for detection. We quite recently reported that suspension culture promoted differentiation toward definitive endoderm more efficiently than adherent culture and that iPS-derived beta cells in suspension culture effectively reversed hyperglycemia in diabetic mice [33, 36], so we differentiated MODY3-iPSCs into pancreatic beta cells using this suspension culture protocol (Fig 4A). qPCR showed that *HNF1A* mRNA expression gradually increased toward pancreatic beta cell differentiation (Fig 4B). We obtained MODY3-iPS beta cell spheroids most of which were 200±50 μm in diameter (Fig 4C). These MODY3-derived beta cells were positive for insulin c-peptide, PDX1, NKX6.1 and HNF1A at the protein level Fig 4D and 4E, indicating these cells had successfully differentiated into pancreatic beta cells.

### P291fsinsC mutant proteins were minimally translated into MODY3-iPS-beta cells

**Fig 5:**
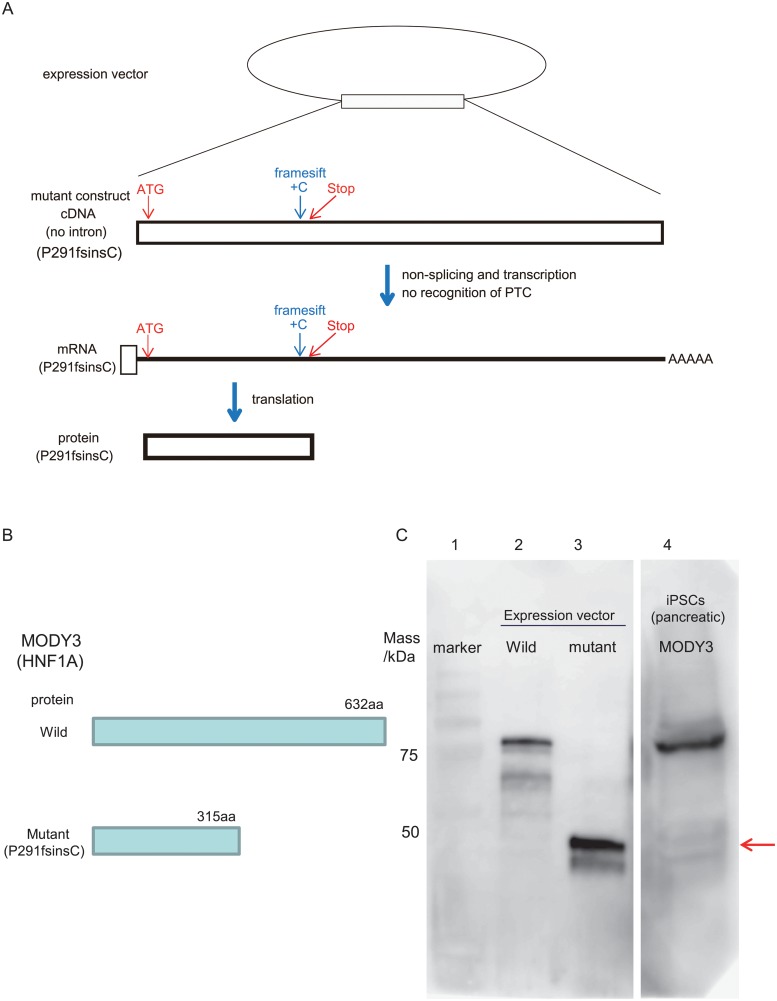
Wild or mutant protein expression of MODY3-iPS-beta cells. (A) Transcriptional and translational schema of P291fsisnC mutant gene artificially constructed in the expression vector. (B) Putative amino acid number for wild and mutant HNF 1A. (C) Western blot analysis of wild or mutant protein expression in vitro or ex vivo. Lane1; molecular marker, lane2; lysates of lentiX 293T cells transfected with wild type HNF1A gene, lane3; lysates of LentiX 293T cells transfected with P291fsinsC HNF1A mutant gene, lane4; pancreatic beta cells differentiated from MODY3-iPS cells. The amount of sample protein loaded on each lane in the gel was as follows: lane2; 1.6 μg, lane3; 0.012 μg, lane4; 80.0 μg. Arrow indicated position of mutant protein band. Predicted band size: wild type; 81 kDa, mutant; 35 kDa.

**Fig 6:**
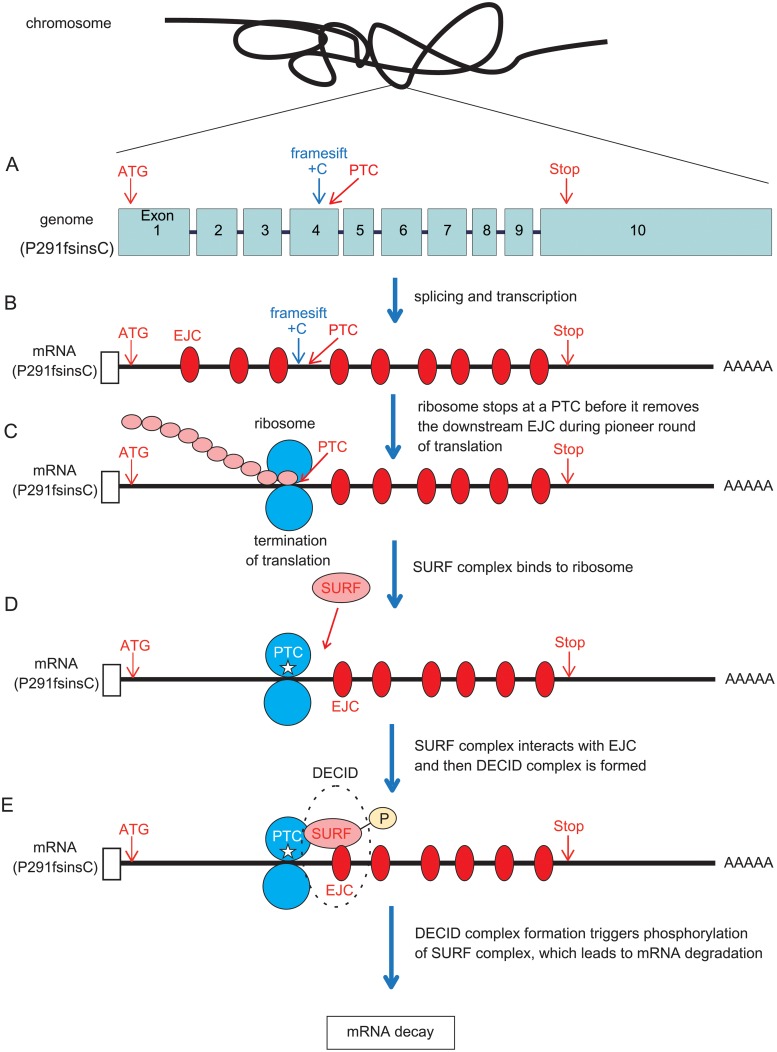
Putative schema of NMD for P291fisnsC mutant mRNA in MODY3-derived beta cells. (A) HNF1A genomic DNA has 10 exons and 9 introns. PTC exists within exon 4, and the original stop codon is located within exon 10. (B) After P291fsisnC mRNA is transcribed from genomic DNA and spliced, 9 exon junction complexes (EJC) are left. (C) During pioneer round of translation of P291fsinsC mutant protein, ribosome stops at a PTC, leaving the downstream EJC. (D) When translation is finished, SURF complex binds to ribosomes. SURF complex interacts with EJC and then DECID complex is formed. (E) DECID complex formation triggers phosphorylation of SURF complex, which leads to mRNA degradation. NMD; non-sense mediated mRNA decay, PTC; premature termination codon, EJC; exon junction complex.

To detect the truncated form of P291fsinsC mutant proteins, we made an artificial construct which contained mutant cDNA for HNF-1a without any intron sequences, as shown in Fig 5A. When mutant mRNA is transcribed from expression vector, splicing doesn’t occur due to the lack of introns. Since PTC of mutant mRNA is recognized as original stop codon, mutant mRNA is supposed to be stabilized, and mutant proteins are stably translated (Fig 5A). On the other hand, in vivo, P291fsinsC mutant mRNA is transcribed from genomic DNA and spliced to remove introns (Fig 6). This splicing is regarded critically important for distinguishing between PTC and the original stop codon. Once PTC is recognized, PTC-bearing mRNA is destroyed by NMD, and mutant protein is minimally translated from mutant mRNA (Fig 6). Therefore, it is generally thought that the amount of mutant protein translated from PTC-bearing mRNA is less than that of wild type protein in vivo.

To compare the amount of mutant and wild protein, we tried to detect those which were translated from expression vectors or genome of MODY3-iPSCs by western blot. P291fsinsC mutant protein was shorter than wild type protein due to C-terminal truncation, but the N-terminal sequence was conserved between wild and mutant proteins (Fig 5B). Hence, we performed western blot analysis using N-terminal region recognizing antibody to detect and distinguish wild (632 aa) and mutant (315 aa) proteins by molecular weight. We were able to detect strong bands of wild and mutant protein in the lysates of LentiX293T cells transfected with wild or mutant *HNF1A* expression vectors, respectively. In contrast there are no bands of mutant proteins in the lysates of pancreatic lineage cells differentiated from MODY3-iPSCs (Fig 5C). We previously reported that establishment of MODY5 (R177X)-iPSCs and R177X mutant RNA was also destroyed by NMD due to PTC [30, 37, 38]. Therefore, we analyzed protein levels for these MODY5-iPSCs by WB in the same way. Definite bands of wild and mutant proteins were detected in transfected cells. On the other hand, in pancreatic lineage cells differentiated from MODY5-iPSCs, a clear-cut signal of wild type protein was detected, but no mutant protein (S1 Fig). These data indicate that P291fsinsC and R177X mutant proteins, both of which were produced from PTC-bearing mRNAs, barely existed in the pancreatic lineage cells differentiated from MODY-iPSCs

## Discussion

In this study, we successfully established MODY3-iPSCs from Japanese patients by using SeV. It is advantageous to use SeV for generating iPS cells because unlike retro or lentivirus, SeV doesn’t have the ability to insert transgenes into a host genome [39, 40] and preservation of the genomic structure is guaranteed for investigating the pathogenesis of hereditary disease. Initial establishment of Caucasian MODY(1, 2, 3, 5 and 8)-iPS cells employed Cre-excisable polycistronic lentivirus [10]. In that system, transgene–free MODY-iPSCs can be obtained by removing the genome-integrated transgenes surrounded by two loxP sites using Cre recombinase. However, 5’ and 3’viral LTR sequences outside the loxP sites cannot be excised, so that these viral LTR sequences remain in the genomes of these MODY-iPSCs even after the removal of the transgenes. Another group established Caucasian MODY2-iPSCs using non-integrating SeV and examined glucokinase activity of the cells [11]. Since the disease gene, HNF1A, of our MODY3-iPSCs is categorized as a transcription factor, while glucokinase in MODY2-iPSCs is categorized as an enzyme, it is suggested that different mechanisms may contribute to the pathophysiology of MODY.

As mentioned earlier, various mutations of several disease genes can develop MODY [16–18]. Possible effects of a mutant protein have been suggested by in vitro experiments [19, 21, 41–45]. In those studies, mutant proteins were examined in various cell lines. including MIN6, HepG2 and Caco-2 cells, and suggested to have decreased, non-functional, or dominant negative activity. However, these expression vector constructs used for in vitro experiments have one critical problem when examining the effects of PTC. Although a 3’-intron flanking exon after PTC sequence and splicing are required for the recognition of PTC in the NMD pathway [27, 28], these expression vector constructs have only one exon; therefore, it remains unclear whether the PTC-bearing mRNA is correctly destroyed by NMD in those assays.

It is estimated that PTC is responsible for 30% of known disease-associated mutations [28, 46]; in the case of MODY3, it is reported that about 30% of mutations have PTC [47]. Importantly, in vitro studies of some truncated proteins have not always correlated with the mutant phenotype in vivo [48]. Additionally, escaping the ablation of PTC-bearing mRNA from NMD causes severe clinical phenotypes, such as peripheral demyelinating neuropathy, central dysmyelinating leukodystrophy, and Waardenburg syndrome, Hirschsprung disease (PCWH), or beta-thalassemia [48, 49]. In the case of EJC-dependent NMD including P291fsinsC and R177X, only one molecule mutant protein can be translated from each mutant mRNA in principle, because pioneer round translation occurs once before mRNA degradation (Fig 6). Therefore, the putative amount of mutant protein should be very small, if any. Given these facts, a proper assay system which examines disruption of transcripts with PTC in the NMD pathway and amount of mutant protein is important for an elucidation of the molecular mechanism of pathogenesis.

In our previous and present research, we investigated the behavior of the mutant genes using MODY-iPS cells: MODY3 (P291fsinsC) and MODY5 (R177X) [30]. Although we found that sequence signals of P291fsinsC and R177X mutant transcripts were weaker than that of wild type transcripts during the differentiation progression from MODY-iPSCs to beta cells, these mutant transcripts increased with CHX treatment (Fig 3), [30], indicating that the degradation of mutant mRNA was brought by NMD. Moreover, we demonstrated by western blot analysis that amounts of these mutant proteins were markedly less than that of wild type proteins in pancreatic lineage differentiated MODY-iPSCs (Fig 5, S1 Fig). In our previously established-MODY5 case, the nonsense mutation of R177X in the *HNF1B* gene produced non-functional or dominant negative protein, dependent on the cell lines used in the in vitro assay [43–45], suggesting that this MODY might be caused by a haplo-insufficiency or a dominant-negative pattern. It was reported that P291fsinsC mutant proteins acted as dominant-negative mutant in vitro assays, and these MODY might be caused by dominant-negative effects [19, 21]. Our data showed that mutant proteins didn’t exist within the detectable range, which indicates very low translation, strongly suggesting mutant mRNA degradation by NMD. From these data, the existence of a dominant negative effect caused by truncated protein seems less likely.

On the other hand, mutant HNF1A mRNA is expressed in hepatic cell as well as pancreatic cell and it is possible that it must also be degraded in hepatic cell by NMD, although we did not examine the expression of mutant mRNA and protein in hepatic lineage cells. In this patient, disfunction of insulin secretion from pancreatic beta cells was observed, but functions of hepatic cells were clinically almost normal. From the clinical standpoint of view, we think some compensation may occur in hepatic cells even if HNF1A mRNA is degraded, which should be proved in the future. Taken together, our results suggest that MODY3 (P291fsinsC) and MODY5 (R177X) are caused by the haplo-insufficiency effect rather than a dominant negative manner.

This approach is useful for studying other genetic diseases caused by a mutant transcript with PTC that is expressed in unavailable tissue like the central nervous system. Differentiating disease specific hiPSCs into previously unavailable tissues enabled us to examine the stability of a mutant mRNA with PTC and to compare the amount of mutant and wild type protein, leading to a more accurate understanding of pathogenesis.

## Supporting information

S1 Fig Wild or mutant protein (HNF1B) expression in pancreatic lineage cells differentiated from MODY5-iPSCs. Western blot analysis of wild or mutant protein expression in vitro or pancreatic lineage differentiated MODY5-iPSCs. Lane1; lentiX 293T cells transfected with expression vector encoding wild type HNF1B gene, lane2; LentiX 293T cells transfected with expression vector encoding R177X HNF1B gene, lane3; pancreatic lineage differentiated MODY5-iPS-beta cell, lane4; molecular marker. The amount of sample protein loaded on each lane in the gel was as follows: lane1; 4 μg, lane2; 8 μg, lane3; 40 μg). Arrow indicated position of mutant protein band. Predicted band size: wild type; 61 kDa, mutant; 20 kDa.
(EPS)
